# Case report of ipsilateral humeral shaft fracture and shoulder dislocation treated by closed reduction followed by intramedullary nailing

**DOI:** 10.1016/j.tcr.2021.100516

**Published:** 2021-08-04

**Authors:** Maya Hara, Ken Yamazaki

**Affiliations:** Orthopedics surgery department, Higashitotsuka Memorial Hospital, 548-7 Shinano-cho Totsuka-ku, Yokohama, Kanagawa, Japan

**Keywords:** Humeral shaft fracture, Anterior shoulder dislocation, Humeral head dislocation, Ipsilateral humeral shaft fracture and shoulder dislocation

## Abstract

Combined humeral shaft fracture and shoulder dislocation injury is rare. Consequently, there are no standard treatment protocols for this injury. We present a case of a 33-year-old man who slipped off a motorcycle and incurred a right humeral shaft fracture and right shoulder dislocation. We performed closed reduction of the shoulder followed by intramedullary nailing. The protocol is different from most in existing papers and results in a good prognosis. However, gentle reduction is essential to avoid iatrogenic neurovascular damage.

## Introduction

**Fig. 1:**
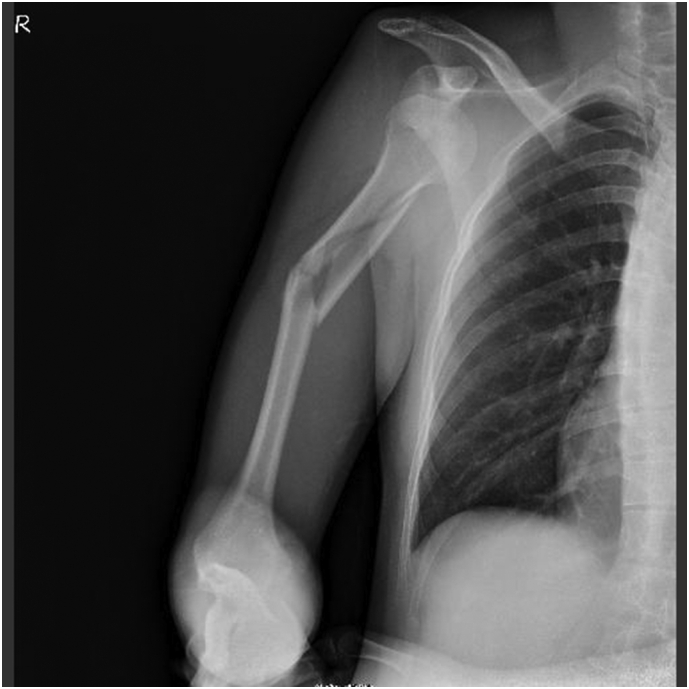
Initial X-ray of the patient.

**Fig. 2:**
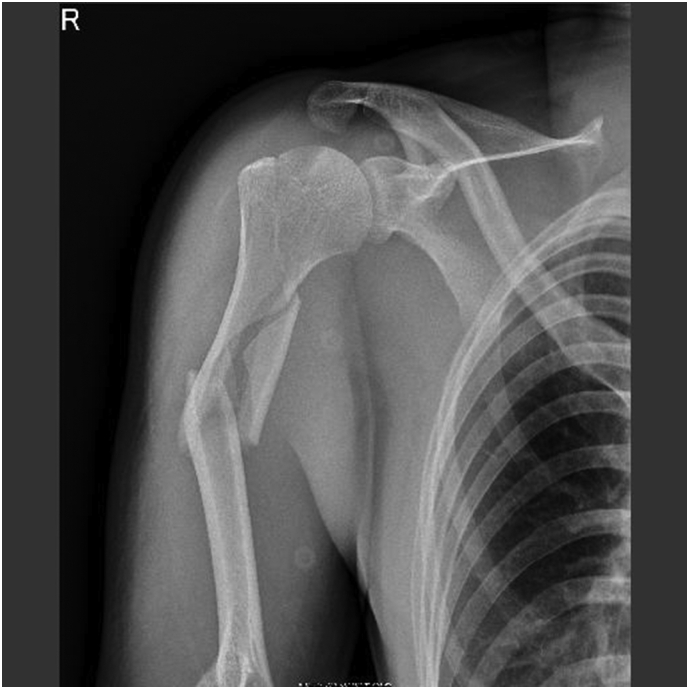
X-ray of after closed reduction.

**Fig. 3:**
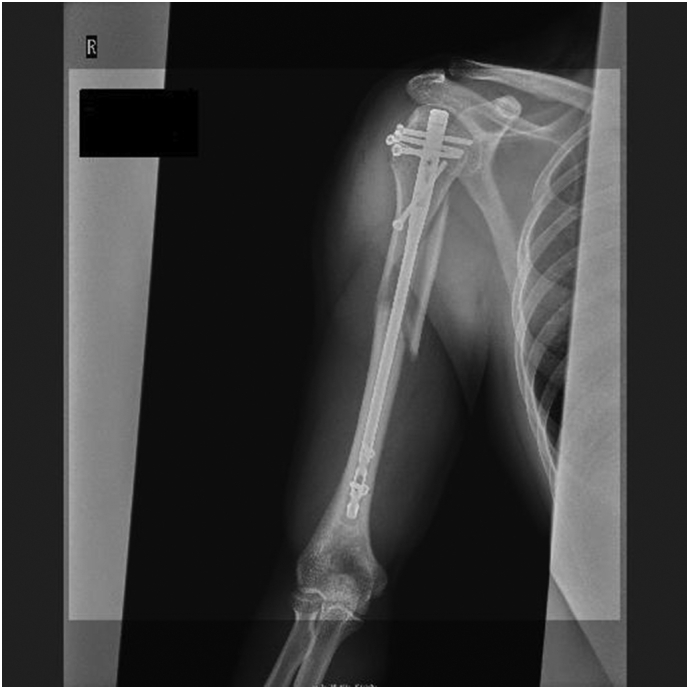
Immediate postoperative X-ray.

**Fig. 4:**
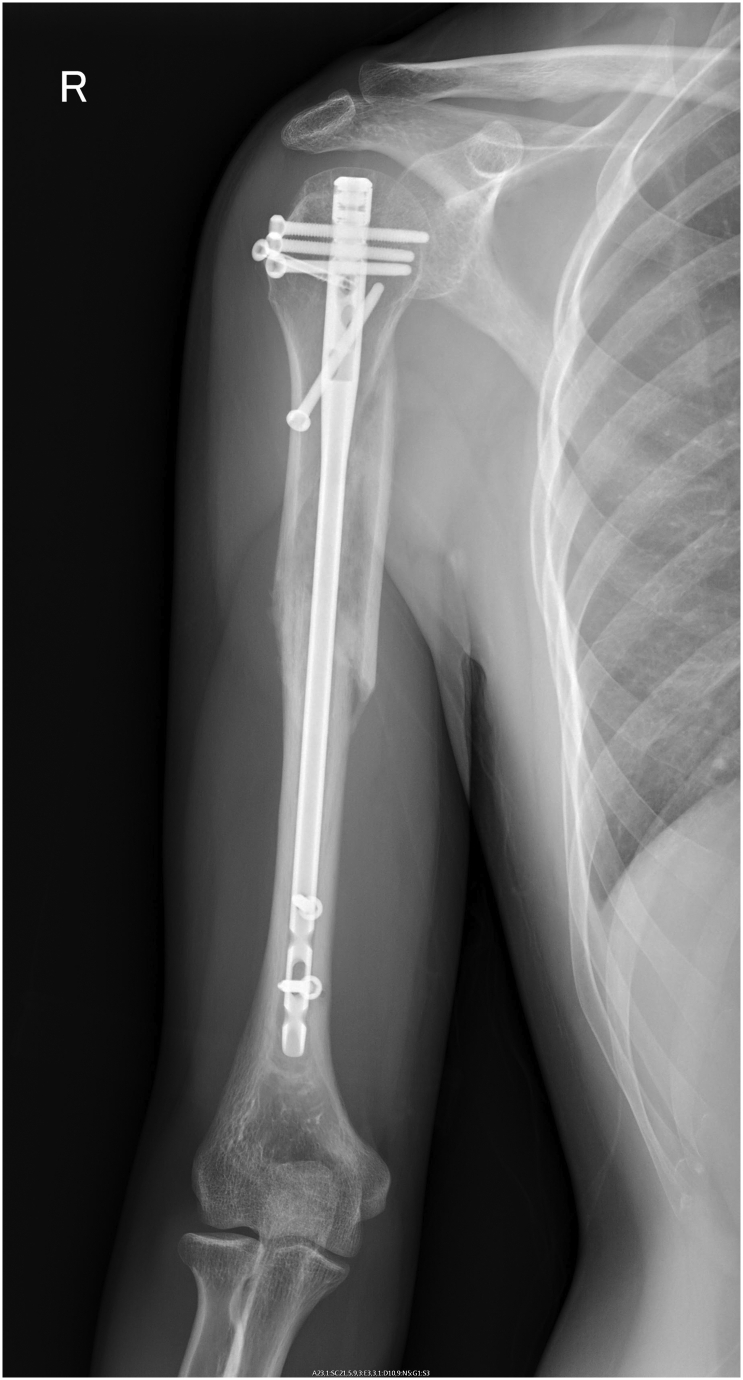
X-ray shows bone union.

Both humeral shaft fracture and shoulder dislocation are common injuries. However, combined humeral shaft fracture with shoulder dislocation is relatively rare. As a result, there are no standard protocols for the treatment of the injury. We present one case of right humeral shaft fracture with shoulder dislocation that was treated differently from those in most existing papers and resulted in a good prognosis (Fig. 1, Fig. 2, Fig. 3, Fig. 4).

## Case presentation

A 33-year-old, right-hand-dominant, Japanese man was brought to the emergency room by ambulance complaining of right upper extremity pain. He was driving a motorcycle and slipped on a maintenance hatch in an attempt to overtake the car in front of him. He was injured on the right side of his body. In the emergency room, he was awake and alert with normal vital signs. Clinical examination showed right upper extremity deformity. The neurovascular exam was intact. Radiographs showed a right humeral shaft fracture and anterior dislocation of the shoulder. We attempted closed reduction under sedation in the emergency room; however, because of his pain and strong muscle contraction, we were not able to reduce the shoulder dislocation. The next day, we transported him to the operating room. Under general anesthesia, we performed a closed reduction and successfully reduced the shoulder. The duration of the maneuver was 1 min. Upon waking, the patient remained neurovascularly intact. Ten days after the closed reduction, we performed open reduction and internal fixation by using intramedullary nailing (Biomet Affixus Natural Nail long, ACE CCS). At the six-month follow-up, his prognosis was good overall. He did not complain of any pain and performed all of his daily activities without any difficulties. His range of motion of the right shoulder was 140 degrees flexion and 30 degrees extension. His UCLA shoulder score was 28 which indicates good result.

## Discussion

Combined humeral shaft fracture with shoulder dislocation is a rare injury. Consequently, there are no standard protocols for its treatment. In terms of deciding whether to perform surgery or conservative treatment, we should consider the shape of the fracture, comorbid injury, and patient conditions. For young adults, surgery may be performed to prevent deformity, nonunion and contraction. Patients who want to return to work early might also be good candidates for surgery. Regarding surgical treatment, if there is radial nerve palsy, plating is likely to be used to expose and observe nerves during surgery. If there is no nerve palsy, nailing should be considered. According to previous papers, to the best of our knowledge, there are five methods of treatment for this combined injury. The treatment methods have the following advantages and disadvantages.

First, the method that we present in this case was performing closed reduction first and nailing afterward. An advantage is that we were able to perform minimally invasive surgery by using nails. In addition, we were able to relieve the pain caused by dislocation immediately. It is difficult to reduce chronic dislocation; as a result, if we reduce dislocation earlier, there are easier and fewer complications. A disadvantage is that during closed reduction, there is a possibility of injuring nerves and vasculature. A previous paper reported that closed reduction of the shoulder caused radial nerve deficit [1].

Second, some authors suggest that plating should be performed first and shoulder dislocation reduced afterwards to avoid neurovascular injury [2]. As pointed out, neurological and vascular injuries are less likely to occur. However, plate fixation may not be possible immediately if the arm is swelling; as a result, while waiting for surgery, the patient has to endure the pain of dislocation. We also need to mention that the more chronic the dislocation is, the more difficult it is to reduce [3]. During surgery, we can observe the radial nerve; however, it is unclear whether exposing the radial nerve in neurovascularly intact cases is meaningful. In addition, plating fixation requires a larger skin incision than nailing. One case report mentioned that the skin did not heal due to postoperative infection [4].

Third, the other method involves treatment with intramedullary nailing prior to closed reduction. An advantage of this procedure is decreasing the chances of nerve and vascular injuries during reduction. In contrast, a disadvantage is that the surgical technique is difficult. A previous case report mentioned that distal locking screws were not applied. In addition, they reported improper positioning of the nail [5].

Fourth, in a previous case report, after fixing the humerus with a splint, the authors performed closed reduction of shoulder dislocation. They performed conservative treatment for the humeral shaft fracture. They mentioned that splinting before closed reduction reduces the possibility of neurovascular damage during reduction. It seems that the range of motion is improved for elderly patients [6]. However, we need to consider whether this method is suitable for young patients in terms of considering possible nonunion, contracture and treatment period.

Finally, in one case report, external fixation was selected because the skin had a degloving injury. After external fixation, reduction of shoulder dislocation was performed [7]. Although neurovascular injury can be prevented, external fixation may not be ideal in terms of rehabilitation and patient convenience in cases without degloving injury or any other skin injury.

## Conclusion

In summary, based on the experience in this case, I would like to present a possible option for the treatment of ipsilateral humeral shaft fracture and shoulder dislocation. In previous papers, only four patients were treated with the same procedure [8], [9]. However, gentle reduction is essential. In our case, an expert with more than 30 years of orthopedic surgery performed the reduction, and the duration of the maneuver was only 1 min. If reduction is difficult, an orthopedic surgeon may withdraw early and consider open surgery to prevent neurovascular injury. Depending on the case and the situation, the treatment method of this case may be considered.

## Declaration of competing interest

None.
